# Formulation and Investigation of CK2 Inhibitor-Loaded Alginate Microbeads with Different Excipients

**DOI:** 10.3390/pharmaceutics15122701

**Published:** 2023-11-29

**Authors:** Boglárka Papp, Marc Le Borgne, Florent Perret, Christelle Marminon, Liza Józsa, Ágota Pető, Dóra Kósa, Lajos Nagy, Sándor Kéki, Zoltán Ujhelyi, Ádám Pallér, István Budai, Ildikó Bácskay, Pálma Fehér

**Affiliations:** 1 Department of Pharmaceutical Technology, Faculty of Pharmacy, University of Debrecen, Nagyerdei Körút 98, H-4032 Debrecen, Hungary; papp.boglarka@pharm.unideb.hu (B.P.); jozsa.liza@pharm.unideb.hu (L.J.); peto.agota@pharm.unideb.hu (Á.P.); kosa.dora@pharm.unideb.hu (D.K.); ujhelyi.zoltan@pharm.unideb.hu (Z.U.); adampaller@gmail.com (Á.P.); bacskay.ildiko@pharm.unideb.hu (I.B.); 2 Institute of Healthcare Industry, University of Debrecen, Nagyerdei Körút 98, H-4032 Debrecen, Hungary; 3 Small Molecules for Biological Targets Team, Centre de Recherche en Cancérologie de Lyon, Centre Léon Bérard, CNRS 5286, INSERM 1052, Université Claude Bernard Lyon 1, Univ Lyon, 69373 Lyon, France; marc.le-borgne@univ-lyon1.fr (M.L.B.); christelle.marminon-davoust@univ-lyon1.fr (C.M.); 4 Univ Lyon, Université Lyon 1, CNRS, INSA, CPE, ICBMS, 69622 Lyon, France; florent.perret@univ-lyon1.fr; 5 Department of Applied Chemistry, Faculty of Science and Technology, Institute of Chemistry, University of Debrecen, Egyetem Tér 1, H-4032 Debrecen, Hungary; nagy.lajos@science.unideb.hu (L.N.); keki.sandor@science.unideb.hu (S.K.); 6 Doctoral School of Pharmaceutical Sciences, University of Debrecen, Nagyerdei Körút 98, H-4032 Debrecen, Hungary; 7 Faculty of Engineering, University of Debrecen, Ótemető Utca 2–4, H-4028 Debrecen, Hungary; budai.istvan@eng.unideb.hu

**Keywords:** protein kinase CK2, CK2 inhibitor DMAT, alginate microbeads, Transcutol^®^ HP, polyvinylpyrrolidone

## Abstract

The aim of this study was to formulate and characterize CK2 inhibitor-loaded alginate microbeads via the polymerization method. Different excipients were used in the formulation to improve the penetration of an active agent and to stabilize our preparations. Transcutol^®^ HP was added to the drug–sodium alginate mixture and polyvinylpyrrolidone (PVP) was added to the hardening solution, alone and in combination. To characterize the formulations, mean particle size, scanning electron microscopy analysis, encapsulation efficiency, swelling behavior, an enzymatic stability test and an in vitro dissolution study were performed. The cell viability assay and permeability test were also carried out on the Caco-2 cell line. The anti-oxidant and anti-inflammatory effects of the formulations were finally evaluated. The combination of Transcutol^®^ HP and PVP in the formulation of sodium alginate microbeads could improve the stability, in vitro permeability, anti-oxidant and anti-inflammatory effects of the CK2 inhibitor.

## 1. Introduction

Protein kinase CK2, also known as casein kinase 2, is a serine/threonine kinase with two catalytic subunits (α) and/or (α’) and two regulatory subunits [1]. CK2 regulates multiple signaling pathways involved in tumor cell survival, proliferation, migration, and invasion [2,3]. The expression and kinase activity of CK2 is elevated in various types of cancers, and increases tumor aggressiveness through the phosphorylation of numerous cytosolic substrates [2,4,5]. 

Elevated CK2 kinase activity is frequently observed in many types of human tumors [6]. The downregulation of CK2 by chemical inhibitors or via genetic approaches promotes cell apoptosis and inhibits tumor cell migration and tumor growth [6]. 

Protein kinase CK2 has been also suggested as a possible target in inflammatory processes and is proposed to have some complex and important roles in the pathogenesis of inflammatory diseases [7,8]. CK2 activity promotes the activation of the NF-kB, PI3K–Akt–mTOR, and JAK–STAT pathways [8]. The protein kinase CK2 is therefore a target for the development of new anti-cancer and anti-inflammatory therapies [7]. 

CK2 activation was also reported to enhance reactive oxygen species production [9]. CK2 plays an important role in the oxidative stress signaling pathway. The anti-oxidant-activated transcription factor, nuclear erythroid factor 2 (Nrf2), regulates the induction of cytoprotective genes against oxidative injuries. Treatment with CK2 inhibitor can block the induction of these endogenous nuclear genes in cells, preventing oxidative damage [10,11]. 

Pagano et al. demonstrated that substituted benzimidazole derivatives are potent inhibitors of CK2 [12]. The substituted heterocyclic structure designed to address the ATP-binding pocket of CK2 allows a diverse derivatization of the ring system. Therefore, numerous benzimidazole derivatives inhibiting CK2 have already been described [13,14]. Due to the poor solubility of the molecule, the development of an efficient drug delivery system was a challenging task. The active ingredient selected for our study was 2-dimethylamino-4,5,6,7-tetrabromo-1*H*-benzimidazole (DMAT). 

Among various natural polysaccharides, alginates have been widely used in the formulation of drug delivery systems for last three decades [15]. Alginate is a copolymer of (1,4)-linked β-D-mannuronate and α-L-guluronate. Alginate-based microparticles as drug delivery systems are biocompatible and biodegradable, and protect the drug from the harsh environmental conditions of the GI tract [16]. Furthermore, they have targeting efficiency, sustainability, and controllable release [17,18,19]. During the formulation of alginate-based microbeads, the active substance is entrapped in a gel of alginate that is crosslinked with divalent cations such as Ca^2+^, Ba^2+^, and Sr^2+^, with uronic acid residues in alginate [20,21,22,23]. 

Transcutol^®^ HP (TC) is a diethylene glycol monomethyl ether that is widely used as a solvent/solubilizer and can improve the solubility of various poorly water-soluble drugs. It is also used as a penetration enhancer in various dosage forms and several authors demonstrated that it could improve in vivo drug absorption, the in vitro dissolution rate and drug release, leading to the improved oral bioavailability of the drug [24,25,26]. 

Polyvinylpyrrolidone (PVP) is a bulky, non-toxic, non-ionic polymer of an amphiphilic nature that is widely used for the formulation of microparticles (MP). It prevents the aggregation of MPs, may serve as a surface stabilizer, and may provide solubility in diverse solvents [27]. The safe use of PVP in pharmaceutical dosage forms has been demonstrated in several articles [28].

The aim of our present study was to prepare CK2 inhibitor-loaded alginate microbeads to enhance the bioavailability of the active ingredient. A potent and selective CK2 inhibitor, DMAT, was selected as an active ingredient, and two different excipients alone and in combination were used in our formulations. TC was chosen to improve the solubility, in vitro drug release and permeability of DMAT. PVP was added to the compositions to prevent drug leaching during preparation and to stabilize our formulations.

In order to characterize the formulations, mean particle size, encapsulation efficiency, and swelling behavior were determined, and scanning electron scanning (SEM) analysis was conducted. The biocompatibility and permeability of our formulations were tested on the Caco-2 adenocarcinoma cell line. In vitro dissolution and enzymatic stability tests were also performed. Finally, the anti-oxidant and anti-inflammatory effects of our formulations were also tested. 

## 2. Materials and Methods

### 2.1. Materials

DMAT (CAS# 749234-11-5) was obtained from Sigma-Aldrich Buchs (St. Gallen, Switzerland). The in silico physicochemical characterization of DMAT was carried out using SwissADME [29]. Low-viscosity-grade sodium alginate was obtained from BÜCHI Labortechnik AG (Flawil, Switzerland). Transcutol^®^ HP (diethylene glycol monoethyl ether) was obtained from Gattefossé (Saint-Priest, France. The human adenocarcinoma cancer cell line (Caco-2) originated from the European Collection of Authenticated Cell Cultures (ECACC, Public Health England, Salisbury, UK). TrypLE™ Express Enzyme (no phenol red) was bought from Thermo Fisher Scientific (Waltham, MA, USA). Calcium chloride dihydrate, 96-well cell culture plates, and culturing flasks were purchased from VWR International (Debrecen, Hungary). Transwell^®^ 24-well cell culture inserts were supplied by Greiner Bio-One Hungary Kft. (Mosonmagyaróvár, Hungary). All other products were purchased from Sigma-Aldrich (St. Louis, MI, USA).

### 2.2. Formulation of CK2 Inhibitor-Loaded Alginate Beads

In order to obtain alginate microparticles, 3.30 g of sodium alginate powder was previously dissolved in 200 mL of distilled water to obtain a 1.5% alginate solution. Due to the hygroscopicity of sodium alginate, a 10–15% excess of the powder should be measured based on the manufacturer’s description. To prepare a 100 mM solution of CaCl_2_, 14.701 g of calcium chloride dihydrate was dissolved in 1000 mL of distilled water. Four compositions containing DMAT (0.5 mg/mL) were prepared (Table 1). In the case of composition 1, DMAT was dissolved in the alginate solution, transferred to a syringe and applied to the appropriate part of the BÜCHI encapsulator B-395 Pro apparat. For composition 2, TC was added, and for composition 3, PVP was added. In the case of composition 4, both excipients were used. According to the manufacturer, the use of highly purified TC is recommended for oral dosage forms. In the production of the microbeads, PVP was dissolved in the hardening solution and TC was dissolved in the alginate solution. 

According to the diameter of the nozzle, the parameters of the encapsulator (liquid flow, vibration frequency, and electrostatic voltage) were set to obtain the correct microparticle chain in the light of a stroboscope lamp (Table 2). A large flat beaker filled with calcium chloride solution (100 mM) was placed under a nozzle on a magnetic stirrer. The alginate beads were left to harden for 15 min in calcium chloride solution. The finely divided particles were washed with distilled water and filtered on a 0.4 µm pore size membrane using a vacuum pump and lyophilized with Scanvac CoolSafe Touch 110-4 Freeze Dryer for 24 h at −110 °C.

### 2.3. Mean Particle Size and SEM

The particle size of the microbeads was determined using a HoribaPartica LA-950V2 laser diffraction particle size analyzer (Horiba, Ltd., Kyoto, Japan). Samples were first diluted 1000×, and then measurements were performed in wet mode. At least five parallel measurements were performed with each sample.

To determine the morphology of particles, SEM analysis was performed on a Hitachi desktop microscope (TM3030 Plus) (Hitachi High-Technologies Corporation, Tokyo, Japan). The instrument is suitable for the direct investigation of the specimens without any surface pre-treatments. Samples were placed on a specific plate with double-sided adhesive and examined using an accelerating voltage of 5 kV [30].

### 2.4. Encapsulation Efficiency

To determine the encapsulated drug content in the beads, a 1 mL sample was taken from the hardening solution (calcium chloride 100 mM) right after formulation. Drug concentration was determined using a UV-VIS spectrophotometer at 420 nm. The amount of entrapped drug was determined from the amount of DMAT remaining free in the hardening solution relative to the amount of the initial drug [31]. The amount of the encapsulated DMAT was calculated using the following formula:

(1) $$\mathrm{Encapsulation}\;\mathrm{efficiency}\left(\mathrm{EE\%}\right)=\frac{\mathrm{amount}\;\mathrm{of}\;\mathrm{initial}\;\mathrm{drug}-\mathrm{amount}\;\mathrm{of}\;\mathrm{free}(\mathrm{not}\;\mathrm{formulated})\mathrm{drug}\left(\mathrm{mg}\right)}{\mathrm{amount}\;\mathrm{of}\;\mathrm{initial}\;\mathrm{drug}\;\left(\mathrm{mg}\right)}\times 100$$

### 2.5. Swelling Behavior

The water sorption behavior of each composition was determined via swelling. The swelling capacity of beads containing DMAT was determined in purified water. One gram from the beads was added to 50 mL of purified water and the dispersions were mixed at 37 °C using a Radelkis OP-912 magnetic stirrer (Radelkis, Budapest, Hungary). For the swelling study, beads were carefully taken out from the water after 24 h, drained with filter paper to remove excess water, and weighed. Weight changes were calculated using the following equation:

(2) $$\mathrm{EWU}=\left(\frac{\mathrm{Ws}-\mathrm{Wd}}{\mathrm{Ws}}\right)\times 100$$

where Ws is the weight of swollen beads and Wd is the initial weight of the dry beads [32].

### 2.6. Enzymatic Stability Test

The study focused on enzymatic degradation using proteolytic enzymes, namely pepsin and pancreatin. An amount of 20 mg of DMAT-loaded particles was introduced into 100 mL of simulated gastric fluid (SGF) containing pepsin or simulated intestinal fluid (SIF) containing pancreatin. The samples were incubated at 37 °C with constant stirring at 100 rpm. The preparation of SGF and SIF followed European Pharmacopoeia specifications. Samples of 1000 µL were collected at predetermined intervals over 120 min, and to stop the enzymatic reaction, an equivalent volume of ice-cold reagent (0.10 M NaOH for SGF and 0.10 M HCl for SIF) was added. Spectrophotometric analysis was then performed at a wavelength of 420 nm [33].

### 2.7. In Vitro Dissolution Study

To investigate the dissolution profile of DMAT-containing microbeads, a USP dissolution apparatus (Erweka, DT 800, Langen, Germany) was used at a 100 rpm paddle speed with 900 mL of dissolution medium at 37 °C. Freshly prepared simulated intestinal fluid (SIF) without pancreatin (pH 6.8) was used as the dissolution medium. During the assay, the dissolution medium (5 mL) was continuously sampled at defined intervals (0, 8 and 24 h) using a syringe. Samples were previously filtered through a 0.45 µm membrane filter and the amount of DMAT was determined using a standard calibration curve. The absorbance of the samples was measured at 420 nm using UV/VIS [34].

### 2.8. MTT Assay

To measure cell viability, an MTT assay was performed on the Caco-2 cell line. Cells were seeded at a density of 10^4^ cells/well in Dulbecco’s DMEM culture media within 96-well plates until complete confluence was achieved. The medium was removed from cells and washed with PBS. Beads containing no active substance and beads containing DMAT were incubated for 1 h at 37 °C with 5% CO_2_. An amount of 100 mg of the samples was dissolved in 10 mL of PBS. After 1 h, the samples were removed from the cells and MTT paint solution (5 mg/mL) was added. The cells were incubated with the solution for 3 h at 37 °C under 5% CO_2_. The paint was then removed from the samples and the resulting formazan crystals were dissolved in a 25:1 mixture of 2-propanol:hydrochloric acid. The yellow tetrazonium salt was converted into purple insoluble formazan crystals by mitochondrial enzymes due to the metabolic activity of the cells. The absorbance of the solutions was measured with a spectrophotometer at a 570 nm wavelength (Thermo-Fisher Multiskan Go (Thermo-Fisher, Waltham, MA, USA), from which the percentage of surviving cells could be calculated. PBS was used as positive control and Triton-X 100 (10% *w*/*v*) was used as a negative control. 

### 2.9. Transepithelial Electrical Resistance Measurement

To determine the membrane integrity of adenocarcinoma cells, transepithelial electrical resistance (TEER) was measured. Cells were seeded at a density of 40^4^ cell/well to form a confluent layer. As a negative control, PBS was used, and as a positive control, Triton X-100 (10% *w*/*v*) was used. Measurement was carried out when Caco-2 cell line monolayers presented TEER values between 1000 and 1200 Ω cm^2^. The cells were incubated with the samples for 1 h with continuous measuring at given intervals during the assay. TEER was measured using a pair of electrodes with Millipore Millicell-ERS 00001 equipment (Merck, Waltham, MA, USA). As a follow-up, measurements were continued in the following 12 h to investigate recovery [35].

### 2.10. In Vitro Permeability Studies

Specific transport studies of all compositions have been performed on the Caco-2 cell line. For the permeability assay, Caco-2 cells were seeded on Transwell^®^ 24-well polycarbonate filter inserts (area: 1.12 cm^2^; pore size: 0.4 μm) at a concentration of 40^4^ cells/insert. TEER was measured before the experiment. 

In transport experiments, we studied the permeability of four formulations containing DMAT via sampling at different intervals. In total, 100 mg of the samples was dissolved in 10 mL of PBS buffer.

The permeability assay was commenced with the addition of 400 µL of the sample solution to the apical chambers of the inserts. A 50 µL aliquot was taken from the apical and basal chamber containing PBS immediately, after 4 h and 24 h. The samples were measured with high-performance liquid chromatography (HPLC) [30].

### 2.11. HPLC Measurements

The HPLC determination of DMAT was performed using the Waters 2695 Separations module equipped with a thermostable autosampler (5 °C), a column module (35 °C), and a Waters 2996 diode array detector (DAD). The separation of the compounds was achieved using a VDSphere PUR C18-M-SE (4.6 × 150 mm, 5 µm) (Agilent technologies, Palo Alto, CA, USA) column. For HPLC-MS measurements, the HPLC instrument was coupled with a MicroTOF-Q-type Qq-TOF MS instrument equipped with an ESI source from Bruker (Bruker Daltoniks, Bremen, Germany). The flow rate and run time were 1.0 mL/min and 12 min, respectively. The active component DMAT was detected with DAD at 260 nm and MS. For the separation of the compounds, isocratic eluent composed of 25% methanol, 40% AcN/water (9/1) containing 0.1% trifluoroacetic acid (TFA), and 35% water was used.

### 2.12. DPPH Anti-Oxidant Test

The DPPH assay is a colorimetric method based on the ability of 2,2-diphenyl-1-picrylhydrazyl (DPPH) to change its dark purple color to yellow in the presence of an anti-oxidant due to its scavenging property. The anti-oxidant scavenging properties of the four formulations containing the CK2 inhibitor were investigated. The test solution was made using DPPH powder (M = 394.33 g/mol) diluted with 96% ethanol. Briefly, 100 µL of each sample diluted in PBS was added to 2 mL of the DPPH test solution (0.06 mM). The reaction mixtures were incubated for 30 min and sheltered from light. As a positive control, Trolox dissolved in PBS (10.0 µM) was used, and as a negative control, 2.0 mL of DPPH solution was used. The quantitative measurement of the remaining DPPH was carried out using the UV spectrophotometer at a wavelength of 517 nm. The anti-oxidant activity percentage (AA%) was determined using the following equation [36]:

(3) $$\mathrm{AA\%}=100-\frac{\left(\mathrm{Abs}\;\mathrm{sample}-\mathrm{Abs}\;\mathrm{blank}\right)\times 100}{\mathrm{Abs}\;\mathrm{control}}$$

### 2.13. Examination of Anti-Inflammatory Effect

The anti-inflammatory effect of the microbeads was investigated on the Caco-2 cell line. Cells were seeded at a density of 10^4^ cells/well on 96-well plates until complete confluency was achieved and the culture medium was removed. The cells were washed with PBS and incubated with the test solutions for 1 h. Briefly, 100 mg of each microbead diluted with PBS was used to perform the test. To induce inflammation, 50 μL of IL-4 (30 ng/mL) was added to the cells and incubated overnight. After incubation, the supernatant was removed from the cells and human TNF-α ELISA Kit (Sigma—RAB0476) was used in accordance with the manufacturer’s instructions. The absorbance of these solutions was measured using Thermo Scientific Multiskan GO microplate spectrophotometer (Thermo-Fisher, Waltham, MA, USA) and was directly proportional to the inflammatory effect of the samples. 

### 2.14. Statistical Analysis

All data were analyzed using GraphPad Prism (version 6; GraphPad Software, San Diego, CA, USA) and are herein presented as means ± SD. A comparison of the results of the swelling behavior, in vitro dissolution test, enzymatic stability assessment, permeability test, TEER measurement, DPPH anti-oxidant test, in vitro anti-inflammatory effect test and MTT cell viability assay was performed with a one-way ANOVA and repeated measures ANOVA followed by Tukey or Dunnett post-testing. The difference in means was considered significant in the case of *p* < 0.05. All experiments were carried out in quintuplicates and repeated at least five times. 

## 3. Results

### 3.1. In Silico Physicochemical Characterization of DMAT

**Figure 1:**
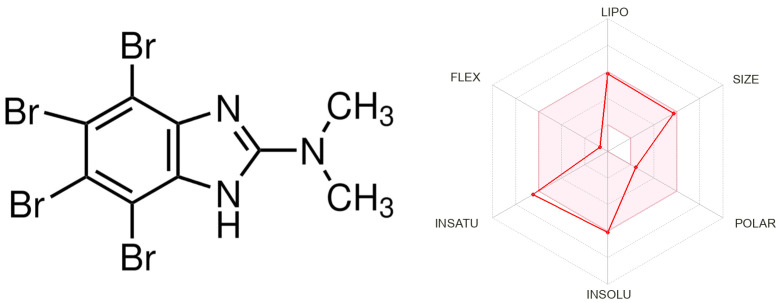
Structure and physicochemical properties of DMAT.

The physicochemical characterization of DMAT (476.79 g/mol) was performed using SwissADME [29]. DMAT has low water solubility and high penetration through the GI tract, but poor penetration through the BBB. Skin penetration occurred at −5.86 cm/s. The Log *P*_o/w_ for DMAT was calculated and the consensus value was equal to 4.12 (the average of five predictions). This consensus value corresponds, for example, to the Log *P*_o/w_ value of the CK2 inhibitor **4p** (5-isopropyl-4-(3-methylbut-2-enyloxy)-5,6,7,8-tetrahydroindeno [1,2-*b*]indole-9,10-dione, consensus Log *P*_o/w_ = 3.91) [37]. Figure 1 shows the structure of DMAT.

### 3.2. Mean Particle Size and SEM

The size of microparticles was calculated using a laser diffraction particle size analyzer and was between 273 (DMAT beads) and 295 µm (beads with both excipients TC and PVP) (Table 3). As shown in Table 3, for each entry, PDI values were between 0.24 and 0.40. Entry 3 (CK2 inhib. beads + PVP) yielded the narrowest size distribution value (PDI = 0.24)

**Figure 2:**
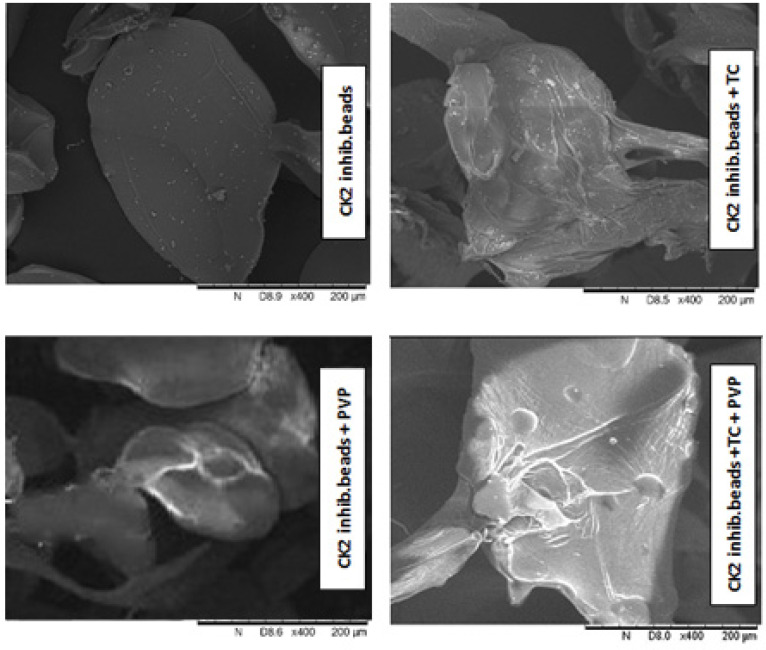
SEM images of the different beads containing DMAT.

Figure 2 shows the image of different dry formulations containing DMAT obtained via SEM. The round morphology of the microparticles was preserved in all four samples; however, the bead structure was damaged in the case of beads containing excipients, presumably due to the high surfactant content. A significant morphological variation was observed when samples containing TC, PVP, or both were compared to the microparticles without excipients. For samples containing the PVP excipient, the difference was clearly visible, and calcium chloride crystals remaining from the hardening solution could be detected in the images. 

### 3.3. Encapsulation Efficiency

The encapsulated DMAT content in the alginate beads was calculated from the equation described in Section 2.4. According to our investigation, the encapsulation efficiency (EE) was in the range of 64 to 84%, as presented in Table 4. There was no significant difference between the DMAT concentrations in composition 2 and 3, which indicated uniform drug dispersion in these formulations. It was justified that there were significant differences in the drug content of the formulation with or without solubilizing excipients. It was observed that composition 4, which contained both TC and PVP, had the highest DMAT content. Comparing the effect of TC and PVP, it can be concluded that the usage of PVP resulted in a higher DMAT content in the beads. 

### 3.4. Swelling Behavior

**Figure 3:**
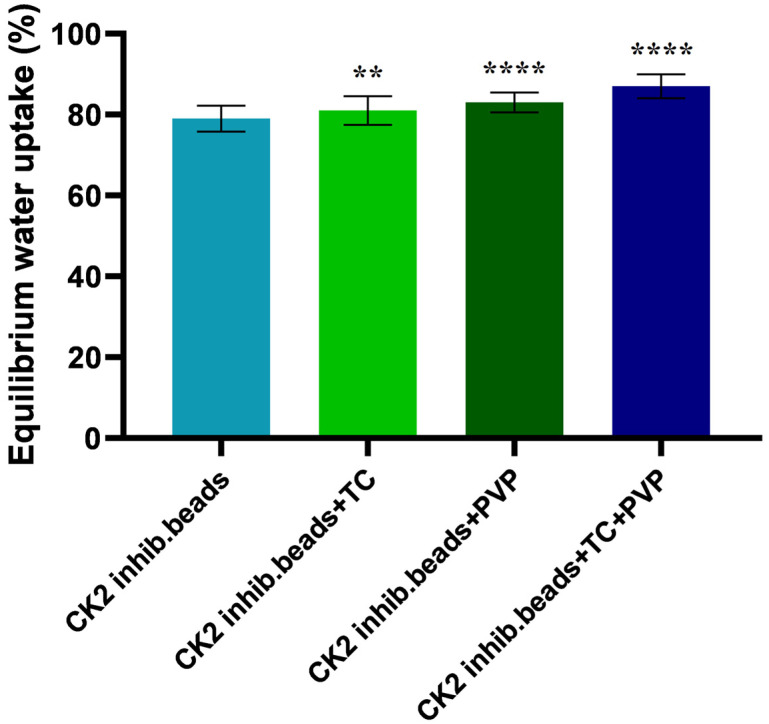
The swelling capacity of the CK2 inhibitor containing beads in distilled water. One gram from the beads was added to 50 mL of distilled water. All of the compositions are represented by the water equilibrium value. Each data point represents the mean ± SD (*n* = 5). An ordinary one-way ANOVA with Dunett’s multiple comparison test was performed to compare the different formulations with excipients with the formulation with DMAT alone. ** and **** indicate statistically significant differences at *p* < 0.05 and *p* < 0.0001.

The equilibrium water uptake of the different compositions was calculated from the equation described in Section 2.5. Figure 3 shows the change in the beads’ weight due to water uptake after 24 h. Alginate (entry 1) and TC excipient + alginate (entry 2) beads showed very similar swelling rates within 24 h (79 and 81%). The water uptake of the PVP excipient containing beads (entry 3) did not significantly increase, as it was 84%. For entry 4, which contained both TC and PVP excipients, the equilibrium water uptake was 89%, which was significantly higher than that for the other compositions. Alginate was mainly responsible for water uptake since, at pH 7, this polymer has the property of swelling, thus increasing its weight.

### 3.5. Result of Enzymatic Stability Test

**Figure 4:**
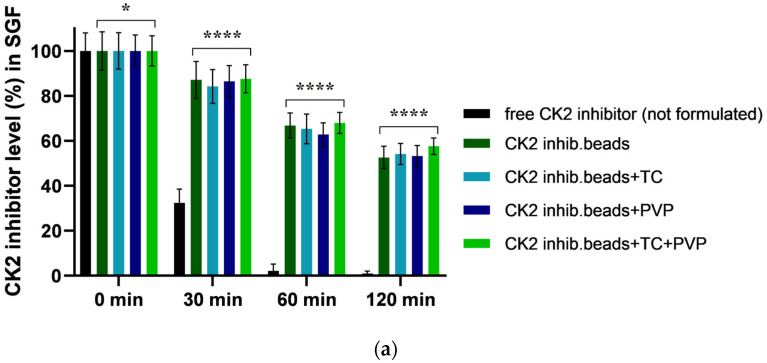

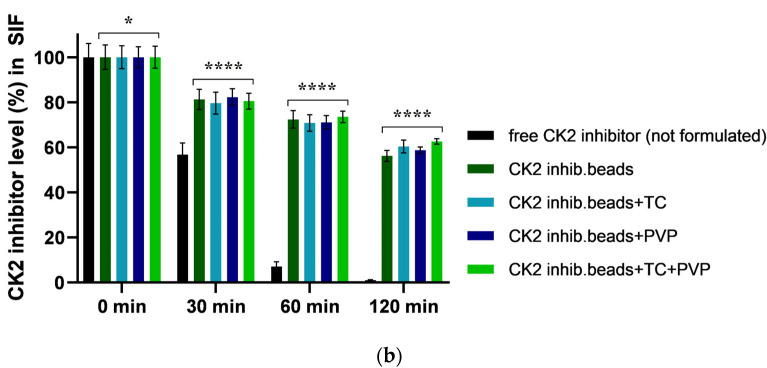
Enzymatic stability of CK2 inhibitor (DMAT)-loaded alginate microbeads in SGF (**a**) and in SIF medium (**b**). Free DMAT (not formulated) was used as a control. Each data point represents the mean ± SD, *n* = 5. Ordinary one-way ANOVA with Dunett’s multiple comparison test was performed to compare the different formulations with excipients with the free (not formulated) DMAT. * and **** indicate statistically significant differences at *p* < 0.01 and *p* < 0.0001.

According to the results, from the free DMAT samples (not formulated), only 2% could be measured in SGF, while 7% could be measured in SIF after 60 min of incubation. The active compound was nearly degraded after 60 min of incubation in SGF, and after 120 min of incubation in SIF. Our experiments revealed that bead formulations are able to protect the active substance. For each formulation, at least 50% of DMAT was protected against degradation by SGF and SIF. Figure 4 depicts the results of this experiment.

### 3.6. In Vitro Dissolution Study

**Figure 5:**
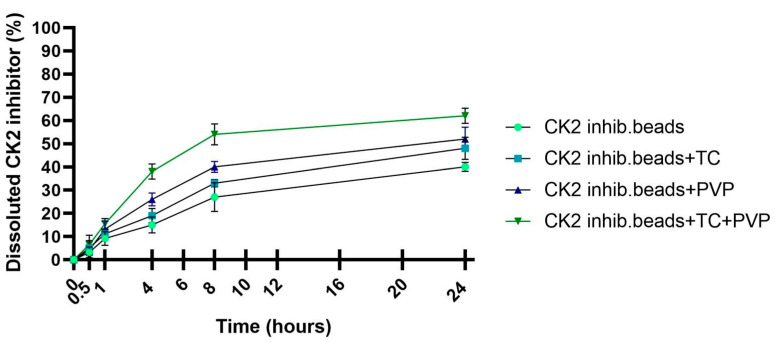
In vitro dissolution profile of CK2 inhibitor form sodium alginate beads in simulated intestinal fluid (SIF) without pancreatin (pH = 6.8). Each data point represents the mean ± SD, *n* = 5.

The CK2 inhibitor release from the different microbeads at pH 6.8 is presented in Figure 5. Our results show that compositions with PVP (entries 3 and 4) had better drug release and dissolution than did compositions without PVP (entries 1 and 2). It was also detected that adding TC to the formulations resulted in better drug release compared with that when adding the formulation that contained only the active agent, DMAT, without excipients. The highest diffused amount of the active substance was from composition 4, which contained both excipients, and DMAT release achieved a value of more than 60% after 24 h. 

### 3.7. MTT Assay

**Figure 6:**
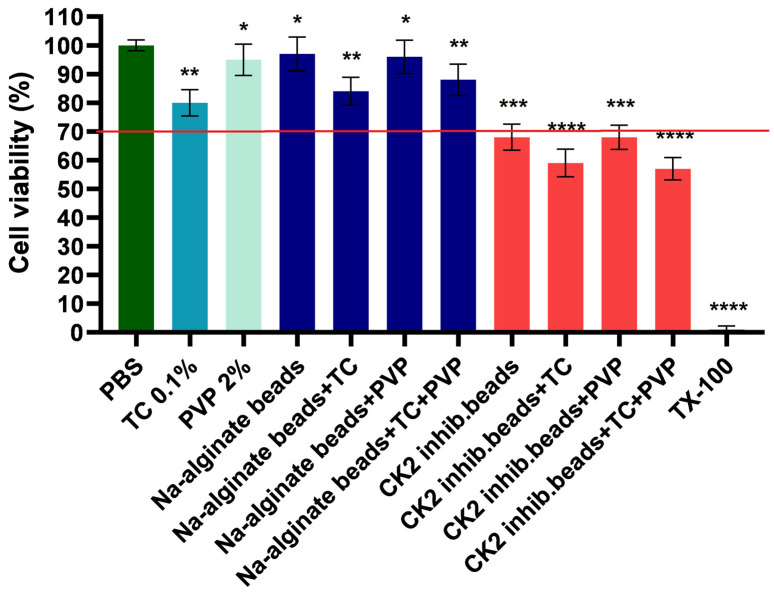
Cell viability test with the MTT assay on Caco-2 cells after incubation with the formulations for 1 h. Cell viability is expressed as the percentage of the negative control (PBS). Each datapoint represents the mean ± SD and *n* = 6. An ordinary one-way ANOVA with Dunett’s multiple comparison test was performed to compare the different formulations with PBS. *, **, *** and **** indicate statistically significant differences at *p* < 0.05, *p* < 0.01, *p* < 0.001 and *p* < 0.0001.

The results of the MTT test are presented in Figure 6. In the experiment, PBS was used as a negative control and Triton X-100 was used as a positive control. Cell viability values were compared to those for PBS and expressed as a percentage of the negative control. For the blank sodium alginate beads, the preparations were found to be safe and well tolerated by the cells, with cell viability consistently exceeding 70% in all cases, which is in accordance with the ISO 10993-5:2009 recommendation [38]. The added excipients were also well tolerated. However, in the case of beads containing DMAT, cell viability was reduced. This reduction in cell viability could be attributed to the presence of the active substance, as empty beads did not show any cytotoxic effect. 

### 3.8. In Vitro Permeability Test

**Figure 7:**
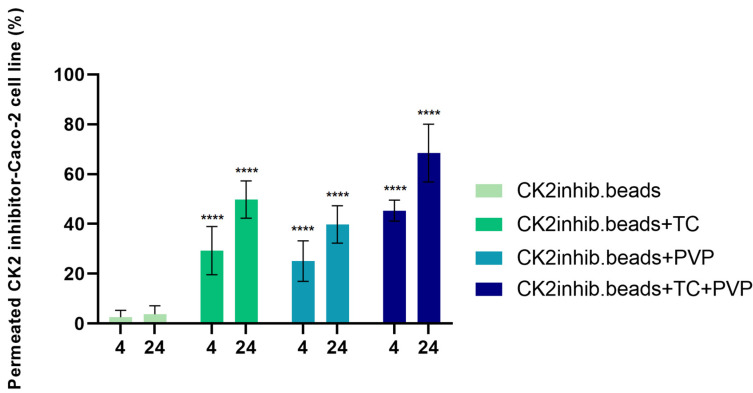
The permeability test of DMAT-loaded alginate microbeads without excipients and with TC and PVP alone and in combination on the Caco-2 cell line. Statistical analysis was performed; **** indicates statistically significant differences at *p* < 0.0001.

The permeability test was performed on the Caco-2 cell line when TEER values reached 800–1000 Ω cm^2^. The amount of permeated components as a function of time is shown in Figure 7. Sampling was performed at 4 and 24 h from both the apical and basolateral parts. Comparing the results to those of the bead without excipients, the highest penetration was obtained for the formulation containing TC and PVP excipients. 

### 3.9. Transepithelial Electrical Resistance Measurements

**Figure 8:**
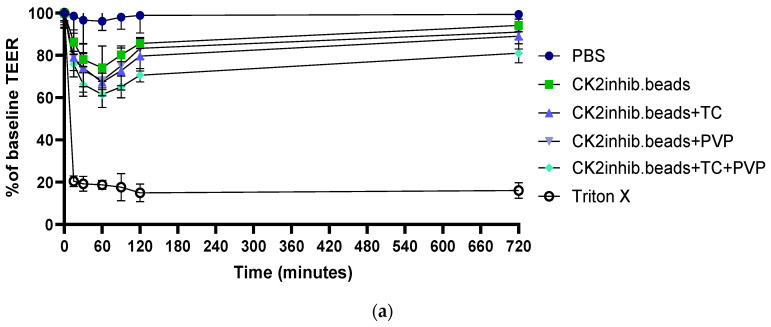

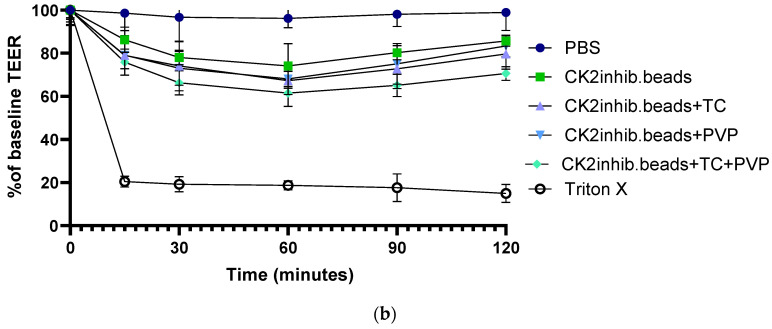
Evaluation of the transepithelial electrical resistance of Caco-2 cells in two contexts: (**a**) across the entire experiment, and (**b**) with a specific emphasis on the initial 120 min. Each data point represents the mean ± SD, *n* = 5.

The membrane integrity of adenocarcinoma cells was determined by TEER measurement. The samples showed a decrease in integrity 15 min after the start of the assay and a steady decrease up to 60 min. After 1 h of treatment, culture medium was added to the cells again, and after 12 h of incubation, the cell integrity had increased to above 90% of the baseline value at the end of the experiment. PBS was used as negative control and (10% *w*/*v*) Triton X-100 as positive control. The sample containing TC and PVP excipients was able to disrupt cell integrity to the greatest extent. Results are shown in Figure 8.

### 3.10. DPPH Scavenging Activity Test

**Figure 9:**
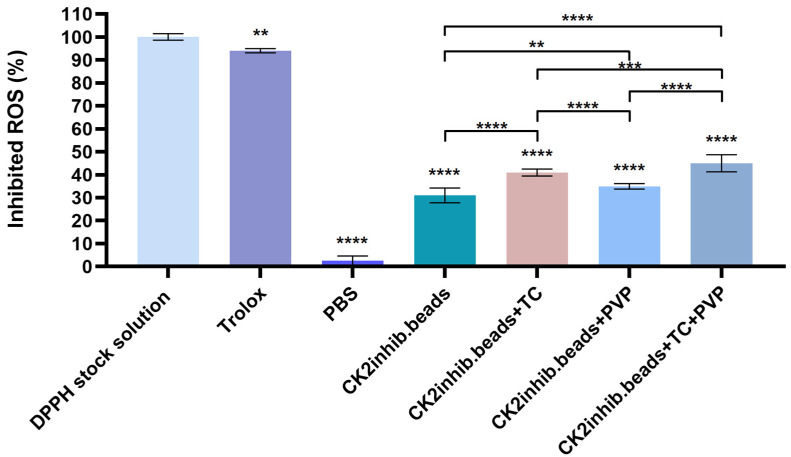
DPPH-scavenging activity of the composition. Data are presented as mean ± SD (*n* = 6). An ordinary one-way ANOVA with Dunett’s multiple comparison test was performed to compare the different formulations with PBS and to compare the formulations with each other. **, *** and **** indicate statistically significant differences at *p* < 0.01, *p* < 0.05 and *p* < 0.0001.

The percentage of the anti-oxidant activity (AA%) of the four formulations was determined using a DPPH test solution. Comparing the four formulations, the microbeads without TC and PVP excipients showed less radical scavenging activity. According to the results, the microbeads containing TC and PVP excipients showed more effective anti-oxidant activity than did the others. The results indicated that DMAT has more effective anti-oxidant activity in the case when microbeads formulated with TC and PVP excipients are used. Figure 9 depicts the results of this experiment.

### 3.11. Examination of In Vitro Anti-Inflammatory Effect

**Figure 10:**
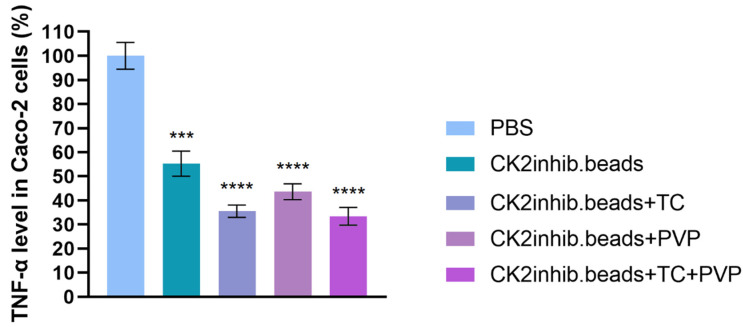
Results of the TNF-α level in the Caco-2 cell line. An ordinary one-way ANOVA with Dunnett’s multiple comparison test was performed to compare the different formulations with PBS. ***, and **** indicate statistically significant differences at *p* < 0.001, and *p* < 0.0001.

The anti-inflammatory effect of the DMAT-loaded microbeads diluted in PBS were conducted by ELISA test on Caco-2 cell line. The negative control was PBS and the mean of the absorbance values was considered as 100% to which the test substances were compared and expressed as a percentage. Figure 10 presents the final results of the anti-inflammatory test on Caco-2 cell line as a percentage of TNF-α level. The results of the study showed that all products were effective in reducing inflammation. The microbeads formulated with TC excipient and the combination of PVP and TC excipients showed the most potent anti-inflammatory effect. The results showed that treatment with DMAT-loaded microbeads resulted in a significant reduction in TNF-α levels on Caco-2 cell line.

## 4. Discussion

The overexpression and important roles of CK2 in several cancers including kidney, lung, head and neck, and prostate, and even in glioblastoma (GBM) have been already reported [2,39]. For instance, CK2 inhibitors induced apoptosis, inhibited tumor cell migration and reduced tumor growth in mouse xenograft models of human GBMs [40,41]. Zheng et al. stated that the CK2α gene is amplified in a large percentage of GBM, and the inhibition of CK2 with CX-4945 (*per os* CK2 inhibitor) inhibits GBM growth in mice.

In our study, we selected the tetrahalogenated CK2 inhibitor DMAT, in order to obtain a proof of concept for the preparation of alginate microbeads. Formulation can be effective for CK2 inhibitors based on an indeniindole scaffold [42]. Then, DMAT-loaded alginate microbeads were formulated via the controlled polymerization method with the Büchi Encapsulator B-395 Pro apparatus. TC was added to the polymer solution in order to improve drug release and to enhance the penetration of DMAT, and PVP was added to the hardening solution to stabilize the formulations [42]. In order to characterize the physical parameters of our microbead formulations, mean particle size was determined.

The size of droplets plays a crucial role in microformulations, as it has the potential to impact both drug release and absorption.

The shape and morphology of the beads were examined via SEM. Among the four formulations, the formulation containing no surfactants retained a regular bead shape, while the formulations containing excipients showed a tailing effect of the beads. According to the literature, this phenomenon often occurs when the amount of surfactants in the formulation is high [43,44]. In order to achieve beneficial properties and adequate toxicity values, slight changes in the bead shape of the surfactant amount were not considered. In light of the cytotoxicity data and with the knowledge of the results of the drug stability studies, the use of high levels of surfactants was indicated.

The microbead formulation containing both TC and PVP excipients showed the highest equilibrium water uptake (89%) compared to that of the other compositions. During formulation, the swelling property is an important factor as swelling behavior influences applicability [45].

The encapsulation efficiency was 64% in the formulation where the CK2 inhibitor was without excipients. Adding PVP to our formulations resulted in a higher CK2 inhibitor content in the beads; the highest value (84%) resulted from those preparations where both TC and PVP were added. PVP improves encapsulation efficiency by preventing the leaching of the drug during preparation. This may be due to PVP increasing the viscosity of the cross-linking solution, so that it can block the pores of the alginate beads and thus prevent the drug’s release into the cross-linking solution [46]. 

According to our in vitro dissolution test, the excipients TC and PVP improved the release profile of the CK2 inhibitor from that in the microbead formulation. The best release value was from the composition containing both excipients as CK2 inhibitor release was more than 60% after 24 h. This greater release of dissolved CK2 inhibitor from the composition could lead to higher bioavailability and absorption [47].

The development of a polymeric and biodegradable matrix prevents active substances from conditions during transit through the gastrointestinal tract [48]. According to our enzyme stability tests, our formulations could successfully shelter the CK2 inhibitor from the harsh environment of the stimulated conditions of the gastrointestinal tract.

The in vitro permeation tests were performed on the Caco-2 cell line. The Caco-2 cell line is derived from colon adenocarcinoma and is widely used as a model of the intestinal epithelial barrier as these cells express similar drug transporters to those in the human intestine [49,50].

Our results showed that sodium alginate microbeads as a carrier system alone could deliver the CK2 inhibitor active agent through the Caco-2 cell line. A study has demonstrated that microcapsules made of alginate could successfully encapsulate and deliver endostatin, an angiogenic inhibitor, to tumor cells [51]. TC improved the permeation of the active substance but the combination of TC and PVP resulted in the highest permeated amount of DMAT through the cell line. Kósa et al. reported that the combination of alginate carriers with amphiphilic surfactants TC and Labrasol improved the absorption of the active substance via the reversible alteration of barrier functions [45].

This study demonstrated that peptide-loaded alginate beads with penetration enhancers play a key role in the bioavailability improvement of the active substance [45]. Mangla et al. formulated nanostructured lipid carriers to enhance the oral delivery of tamoxifen and sulforaphene [52]. Those formulations that contained TC resulted in a better in vitro and ex vivo drug release profile as well as better intestinal permeability [52]. To select the appropriate concentrations of the used excipients, a preliminary viability study was carried out. The concentrations used proved to be the highest that were still tolerable by Caco-2 cells.

TC may improve the bioavailability of active substances in formulations. Hashemzadeh et al. revealed that the efficiency of diethylene glycol monoethyl ether as a solubilizer/penetration enhancer depends on its performed concentration alone or in combination with other excipients [24].

PVP in drug delivery systems has been shown to enhance the drug circulatory time in plasma. Several studies showed that PVP has the longest circulation lifetime among various polymers and its tissue distribution was extremely restricted [53].

The cytotoxic effect of the excipients is a crucial point in the formulations. In order to see the biocompatibility of our formulations, an MTT assay on the Caco-2 cell line was performed. The results showed that the empty sodium alginate beads with and without the excipients TC and PVP were safe and well tolerated by the cells as the cell viability was over 70% in each case. For beads containing DMAT, the cytotoxicity values remained below 70%, which was expected considering the effect of the compound.

DPPH scavenging activity of the four compositions was determined in order to see the anti-oxidant effect of our formulations. DMAT-loaded microbead formulation without excipients showed that DPPH scavenging activity was at 31%; however, those formulations that contained the excipients TC or PVP alone or in combination had a better anti-oxidant effect (41%, 35% and 45%). These results are in accordance with the in vitro dissolution studies, as the excipients could improve the dissolution of DMAT, and a higher amount of the active ingredient resulted in a higher anti-oxidant effect. Thus, the anti-oxidant effect of the formulation depended on the amount of active ingredient dissolved. Several authors demonstrated that TC can improve in vivo drug absorption, the in vitro dissolution rate and drug release, leading to the improved oral bioavailability of the drug [24,25,26]. Spaglova et al. reported that TC improved the solubility and the drug release properties of indomethacin, for example [54].

Several diseases—in which an inflammatory response plays an important role—have been reported to be associated with aberrant CK2 signaling (e.g., breast cancer, glomerulonephritis, and T cell lymphoma) [7,55]; thus, CK2 has been proposed as a target in inflammatory diseases [7].

Wang et al. demonstrated that the CK2 inhibitor (PD144795) inhibited TNF-α-induced p65 phosphorylation on HeLa cells [56]. The anti-inflammatory effect of the DMAT-loaded microbeads were studied using an ELISA test on the Caco-2 cell line. The level of TNF-α, a classic pleiotropic pro-inflammatory cytokine, was determined in anti-inflammatory experiments [57]. CK2 microbead formulation without excipients slightly reduced the level of TNF-α (55%). After adding the excipients TC and PVP to the formulations, higher anti-inflammatory effects were detected (36% and 44%, respectively). The microbeads with both excipients showed the highest anti-inflammatory effect (33%). According to our previous study, using the TC excipient in SNEDDS formulations containing curcumin could improve the anti-inflammatory effect of formulations [58]. Also, these results demonstrated that the excipients influenced in vitro drug release from the formulations, and then modulated the anti-inflammatory effect.

Our results showed that we developed and optimized the formulation of DMAT-loaded alginate microbeads with the efficient help of TC and PVP excipients. Sodium alginate microbeads as a carrier system were proven to prevent the active substance from enzymatic degradation and could improve the permeation of DMAT through the Caco-2 cell-line. The excipient PVP stabilized our formulations and the excipient TC could improve the permeation of active substance through the Caco-2 monolayer. Our formulations also showed good anti-oxidant and anti-inflammatory effects, which were also influenced by the excipients.

## 5. Conclusions

In the present study, alginate microbeads loaded with DMAT were designed with different excipients. The combination of TC and PVP in the formulations improved enzymatic stability and the in vitro dissolution of active ingredient, and also enhanced the permeability of the CK2 inhibitor on the Caco-2 cell line. The in vitro anti-oxidant and anti-inflammatory effect of our formulations were also proven. These results demonstrate that alginate beads may be a promising delivery system for CK2 inhibitors but also point out that choosing the appropriate excipients is a crucial point in formulation.

The role of CK2 and its overexpression is already demonstrated in different types of solid tumors. Furthermore, several CK2 inhibitors are now available and have been shown to be effective against cancers in vitro, in vivo and also in pre-clinical studies. In the future, in vivo animal experiments should be planned, with our formulations, that would prove the relevance of these preparations in cancer therapy.

## Figures and Tables

**Table 1:** Diverse formulations of CK2 inhibitor-loaded alginate beads.

Entry	Composition	Excipient
1	CK2 inhib. beads	-
2	CK2 inhib. beads + TC	Transcutol^®^ HP (0.01% *v*/*v*)
3	CK2 inhib. beads + PVP	PVP (2% *w*/*v*)
4	CK2 inhib. beads + TC + PVP	Transcutol^®^ HP (0.01% *v*/*v*)PVP (2% *w*/*v*)

**Table 2:** The applied parameters of the encapsulator.

Diameter of the Nozzle [μm]	Vibration Frequency [Hz]	Electrostatic Voltage [V]	Flow Rate (mL/min)
200	1800	1000–1200	5.06

**Table 3:** Mean particle size and polydispersity index (PDI) values of the different DMAT-loaded alginate beads. Values are expressed as mean ± S.D., *n* = 5.

Entry	Composition	Particle Size (µm)	PDI
1	CK2 inhib. (DMAT) beads	272.62 ± 10.03	0.40 ± 0.03
2	CK2 inhib. beads+ TC	279.67 ± 10.49	0.38 ± 0.02
3	CK2 inhib. beads + PVP	288.91 ± 5.28	0.24 ± 0.01
4	CK2 inhib. beads + TC + PVP	294.83 ± 8.46	0.33 ± 0.04

**Table 4:** Encapsulation efficiency of the different compositions containing DMAT.

Entry	Composition ^a^	EE (%) ^b^
1	CK2 inhib. (DMAT) beads	64.32 ± 0.72
2	CK2 inhib. beads + TC	70.12 ± 0.81
3	CK2 inhib. beads + PVP	72.35 ± 0.66
4	CK2 inhib. beads + TC+ PVP	84.07 ± 1.02

^a^ Microbeads containing both TC and PVP showed the highest EE% of all the formulations. ^b^ Each data point represents the mean ± SD; *n* = 5.

## Data Availability

The data that support the findings of this study are available from the corresponding author (feher.palma@pharm.unideb.hu) with the permission of the head of the department, upon reasonable request.

## Sample Availability

Samples of the compounds are available from the authors.
